# Species composition, larval habitats, seasonal occurrence and distribution of potential malaria vectors and associated species of *Anopheles *(Diptera: Culicidae) from the Republic of Korea

**DOI:** 10.1186/1475-2875-9-55

**Published:** 2010-02-17

**Authors:** Leopoldo M Rueda, Tracy L Brown, Heung Chul Kim, Sung-Tae Chong, Terry A Klein, Desmond H Foley, Assaf Anyamba, Matthew Smith, Edwin P Pak, Richard C Wilkerson

**Affiliations:** 1 Division of Entomology, Walter Reed Army Institute of Research, Silver Spring, MD 20910-7500, USA; 2 Walter Reed Biosystematics Unit, WRAIR, Museum Support Center, MRC 534, Smithsonian Institution, 4210 Silver Hill Road, Suitland, MD 20746-2863, USA; 3 5th Medical Detachment, 168th Multifunctional Medical Battalion, 65th Medical Brigade, Unit 15247, APO AP 96205-5247, USA; 4 Force Health Protection and Preventive Medicine, 65th Medical Brigade/USAMEDDAC-Korea, Unit 15281, APO AP 96205-5281, USA; 5 Biospheric Sciences Branch, Code 614.4, NASA Goddard Space Flight Center, Greenbelt, MD 20771-2400, USA

## Abstract

**Background:**

Larval mosquito habitats of potential malaria vectors and related species of *Anopheles *from three provinces (Gyeonggi, Gyeongsangbuk, Chungcheongbuk Provinces) of the Republic of Korea were surveyed in 2007. This study aimed to determine the species composition, seasonal occurrence and distributions of *Anopheles *mosquitoes. Satellite derived normalized difference vegetation index data (NDVI) was also used to study the seasonal abundance patterns of *Anopheles *mosquitoes.

**Methods:**

Mosquito larvae from various habitats were collected using a standard larval dipper or a white plastic larval tray, placed in plastic bags, and were preserved in 100% ethyl alcohol for species identification by PCR and DNA sequencing. The habitats in the monthly larval surveys included artificial containers, ground depressions, irrigation ditches, drainage ditches, ground pools, ponds, rice paddies, stream margins, inlets and pools, swamps, and uncultivated fields. All field-collected specimens were identified to species, and relationships among habitats and locations based on species composition were determined using cluster statistical analysis.

**Results:**

In about 10,000 specimens collected, eight species of *Anopheles *belonging to three groups were identified: Hyrcanus Group - *Anopheles sinensis*, *Anopheles kleini*, *Anopheles belenrae*, *Anopheles pullus*, *Anopheles lesteri*, *Anopheles sineroides*; Barbirostris Group - *Anopheles koreicus*; and Lindesayi Group - *Anopheles lindesayi japonicus*. Only *An. sinensis *was collected from all habitats groups, while *An. kleini, An. pullus *and *An. sineroides *were sampled from all, except artificial containers. The highest number of *Anopheles *larvae was found in the rice paddies (34.8%), followed by irrigation ditches (23.4%), ponds (17.0%), and stream margins, inlets and pools (12.0%). *Anopheles sinensis *was the dominant species, followed by *An. kleini, An. pullus *and *An. sineroides*. The monthly abundance data of the *Anopheles *species from three locations (Munsan, Jinbo and Hayang) were compared against NDVI and NDVI anomalies.

**Conclusion:**

The species composition of *Anopheles *larvae varied in different habitats at various locations. *Anopheles *populations fluctuated with the seasonal dynamics of vegetation for 2007. Multi-year data of mosquito collections are required to provide a better characterization of the abundance of these insects from year to year, which can potentially provide predictive capability of their population density based on remotely sensed ecological measurements.

## Background

*Anopheles *mosquitoes of the Republic of Korea (ROK) belong to subgenus *Anopheles *in three groups, namely Hyrcanus, Barbirostris and Lindesayi. The Hyrcanus Group comprises about 30 species worldwide, of which six species are known in the ROK, namely *Anopheles belenrae, Anopheles kleini, Anopheles sinensis*, *Anopheles sineroides*, *Anopheles pullus *and *Anopheles lesteri *[1,2]. The other two species belong to the Barbirostris Group (*Anopheles koreicus*) and the Lindesayi Group (*Anopheles lindesayi japonicus*) [3]. Preliminary data suggest that *An. pullus *and *An. kleini *are the primary vectors of *Plasmodium vivax *malaria near the demilitarized zone (DMZ), while *An. sinensis *is a secondary vector [4]. Females of *An. sineroides *and *An. belenrae *have also been found positive for *P. vivax *by enzyme-linked immunosorbent assay (ELISA) and polymerase chain reaction (PCR), respectively (TAK, unpublished data). *Anopheles lesteri *(= *An. anthropophagus*) is a major vector of malaria in China [5]; however, its vectorial capacity is unknown in the ROK. The other remaining two *Anopheles *species are not considered to be malaria vectors in the ROK [4].

Recent studies [2,5,6] have reported occurrence data of mosquito species from different areas in the ROK. In this study, we conducted comprehensive monthly (May to October) larval collections from selected habitats at three distant locations (Munsan, Hayang and Jinbo). The objectives of this study were to determine the species composition, habitats, seasonal occurrence and geographic distributions of members of the Hyrcanus Group and other group-species from representative areas in the ROK. Monthly satellite-derived normalized difference vegetation index (NDVI) data for the collection sites were compiled to determine the seasonal patterns of larval abundance from various habitats in relation to the background ecological conditions. Previous studies [7-11] have shown that the emergence of various disease vectors and pests including mosquitoes, rodents and locust, tends to follow the flush green vegetation. Therefore, monitoring the ecological conditions can provide valuable information on mosquito population dynamics for use in ecological niche modeling

## Methods

### Specimen collection and identification

**Figure 1:**
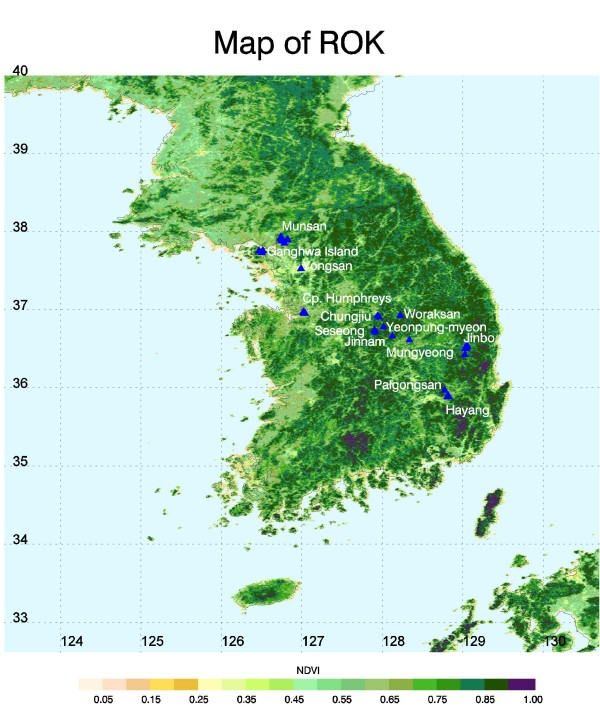
**Map of the Republic of Korea showing the major collection locations of *Anopheles (Anopheles) *species**. NDVI, normalized difference vegetation index.

**Figure 2:**
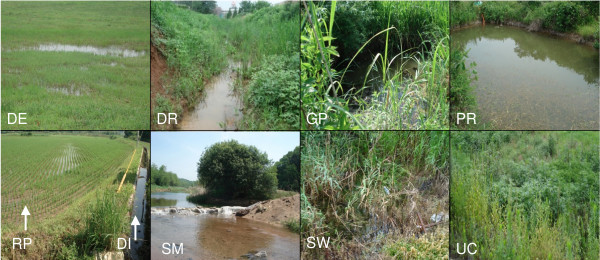
**Larval habitats of *Anopheles (Anopheles) *species**. Ground depressions, DE; irrigation ditches, DI; drainage ditches, DR; ground pools, GP; ponds, PR; rice paddies, RP; stream margins, inlets and pools, SM; swamps, SW; and uncultivated fields, UC.

Mosquito collections of all species were conducted from 15 locations in Gyeonggi, Gyeongsangbuk, and Chungcheongbuk Provinces (including Inchon and Seoul Metropolitan Areas), ROK from May to October 2007 (Figure 1) from various larval habitats (Figure 2). Depending on the habitats, larvae were collected using a standard larval dipper (350 ml, 13 cm diameter) or a white plastic larval tray (25 × 20 × 4 cm) (BioQuip, Rancho Dominguez, CA). Each habitat within a location was surveyed for up to 1-hr or until about 100 larvae were collected. The latitude and longitude of each location was recorded using a hand held Global Positioning System (GPS) unit (Garmin International, Olathe, KS) set to the WGS84 datum. Sampling locations were photographed using a digital camera to assist in verifying the accuracy of the habitat description. Collected larvae were placed in plastic Whirl-Pak^® ^bags (118 ml, 8 × 18 cm) (BioQuip, Rancho Dominguez, CA) filled approximately 1/2 full with water from the collection site. The Whirl-Pak^® ^was then tightly closed to retain air, placed in a cooler, and brought to the laboratory where most larvae were directly preserved in 100% ethanol for molecular identification; the remaining larva were individually link-reared to adult stage, as morphological voucher specimens for this work. Emergent adults were pinned on paper points, each given a unique collection number, and identified using diagnostic morphological characters [1,3]. For molecular species identification, DNA was isolated from individual larval mosquitoes and adults (1 or 2 legs per adult) by phenol-chloroform extraction, and direct sequencing was carried out as described in Wilkerson *et al *[12]. The rDNA ITS2 was amplified using conserved sequence found in the 5.8S subunit, ITS2 forward (5'-TGTGAACTGCAGGACACATGAA-3') and in the 28S subunit, ITS2 reverse (5'-ATGCTTAAATTTAGGGGGTAGTC-3') [13]. PCR products were directly sequenced using Big Dye 3.0 (Applied Biosystems Inc. - ABI, Foster, CA) with an ABI 3100 sequencer (ABI). The sequence was then edited and analyzed using Sequencher (v 4.8, AB). Sequences of *An. sinensis*, *An. lesteri*, *An. pullus*, *An. belenrae *(unknown number 1) and *An. kleini *(unknown number 2) are those of Wilkerson *et al *[12] and Li *et al *[14] using the primers therein. GenBank accession numbers for the above are in Wilkerson *et al *[12] and Li *et al *[14]. Direct sequencing was carried out for about 8,000 samples. Voucher specimens and collection records were deposited in the U.S. National Museum of Natural History, Smithsonian Institution, Suitland, MD.

### Relationship among habitats and locations based on the mosquito species composition

**Figure 3:**
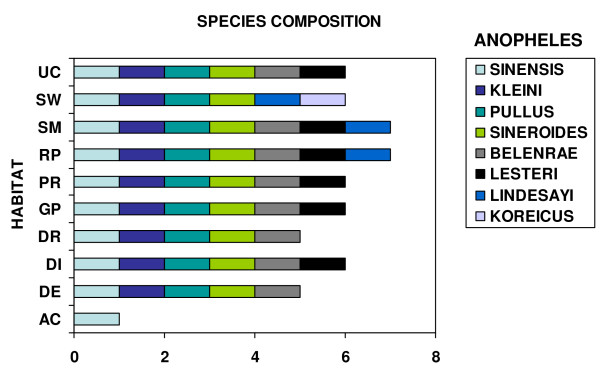
**Relationship between larval habitats and *Anopheles (Anopheles) *species composition in the Republic of Korea**. Artificial containers, AC; ground depressions, DE; irrigation ditches, DI; drainage ditches, DR; ground pools, GP; ponds, PR; rice paddies, RP; stream margins, inlets and pools, SM; swamps, SW; and uncultivated fields, UC.

Relationships among the 10 mosquito positive habitats based on species composition (Figure 3) were investigated using McQuitty (WPGMA) cluster analysis [15]. Sample data from 1-8 of the 10 habitat types at each of 15 selected locations over six months were analysed. Following the approach of Savage *et al *[16], variables representing habitat and location were combined into a single variable (called "habitat site") to perform cluster analysis. A total of 40 positive sites were included in the analysis. For each habitat-pair, the Dice distance coefficients (DCO) were calculated to provide a basis for cluster. DCO is a coefficient based on the presence or absence of taxa, which emphasizes similarity or common taxa [17,18]. Cluster analysis was performed on a distance matrix with values calculated by subtracting each DCO value from 1.0. The specific locations have the following abbreviations (in parenthesis): Camp Humphreys (CH), Chungju, 7 km W (CU), Ganghwa Island (GI), Hayang (HA), Highway 517, 5 km S (HW), Jinbo (JI), Jinnam (JR), Mungyeong, outside (MS), Munsan (MU), Palgongsan Provincial Park (PA), Seseong (SA), Yongsan Park, Seoul (SE), Woraksan National Park (WO), Yeonpung (YE). The habitats have the following abbreviations (in parenthesis): artificial containers (AC), ground depressions (DE), irrigation ditches (DI), drainage ditches (DR), ground pools (GP), ponds (PR), rice paddies (RP), stream margins, inlets and pools (SM), swamps (SW), and uncultivated fields (UC) (Figure 2). The grid coordinates, elevations and related information of above locations are lodged in the WRBU MosquitoMap website, code KSK [19].

### Seasonal occurrence of *Anopheles *species from three selected locations

Monthly larval surveys of *Anopheles *species were conducted from three locations, namely Munsan (MU, Gyeonggi Province), Jinbo (JI, Gyeongsangbuk Province), and Hayang (HA, Gyeongsangbuk Province) (Figure 1). The larval sampling techniques (using larval dipper and tray), as mentioned above, were used to collect larvae from various habitats (AC, DE, DI, DR, GP, PR, RP, SM, SW, and UC). For each above location, the same habitats (collection sites) were sampled for mosquito larvae monthly from May through October.

### Relationship between normalized difference vegetation index (NDVI) data and mosquito larval population densities from various habitats in three locations

The NDVI data used in this study were derived from the atmospherically corrected 8-day surface reflectance Moderate Resolution Imaging Spectroradiometer (MODIS) product MOD09A2 [20], which has a spatial resolution of one kilometer. In order to derive monthly near cloud-free data, we combined the original 16-day data into ~32-day data to create monthly maximum-value composites for 2007 [21]. These monthly data sets were required to compare against mosquito species data that were collected on a monthly time step. Monthly NDVI anomalies were calculated by subtracting the monthly composite data for the 2007 growing season from their respective long-term means covering the period 2000-2008 to determine if there were any site differences from long-term ecological patterns.

Mosquito larval density data were grouped and totaled according to proximity to each other resulting in 15 locations with numerous unique collection sites. Of the 15 locations mentioned above, only three (Hayang, Jinbo, and Munsan) contained complete records for the six months (May through October). The location analyses for NDVI and anomaly values were restricted to these three (Figure 1). A three pixel by three pixel window surrounding each location was used to extract NDVI values. NDVI values were then compared to *Anopheles *larval densities.

## Results and discussion

### Species composition

**Figure 4:**
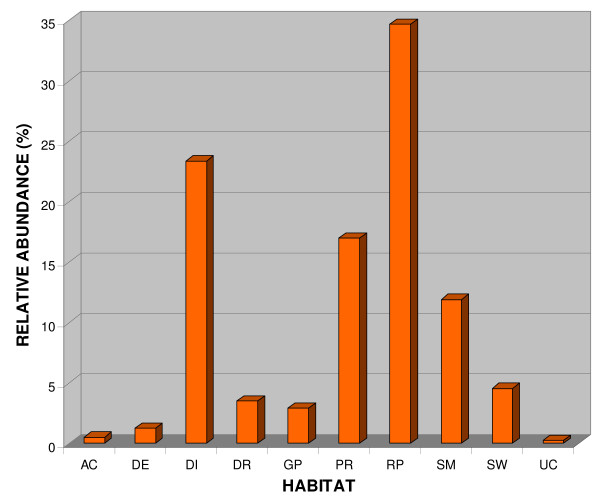
**Relative abundance of *Anopheles (Anopheles) *larvae from various immature habitats in the Republic of Korea**. Artificial containers, AC; ground depressions, DE; irrigation ditches, DI; drainage ditches, DR; ground pools, GP; ponds, PR; rice paddies, RP; stream margins, inlets and pools, SM; swamps, SW; and uncultivated fields, UC.

A total of about 285 batches of *Anopheles *larval samples were collected from different locations in the ROK (Figure 1), and about 10,000 larvae were retrieved and identified to species by PCR assays (2,000 larvae) and sequences (8,000 larvae). Eight species of *Anopheles *were found from various habitats in those locations. As previously reported [2,3], the species belong to three different groups, namely: the Hyrcanus Group species - *An. belenrae, An. kleini, An. sinensis*, *An. sineroides*, *An. pullus*, and *An. lesteri*; the Barbirostris Group - *An. koreicus*, and the Lindesayi Group - *An. lindesayi japonicus*. Figure 3 shows the species composition of the *Anopheles *collected from ten major habitats (AC, DE, DI, DR, GP, PR, RP, SM, SW, and UC). The higher numbers of species (seven out of eight) were collected from the rice paddies, and stream margins, inlets and pools. With the exception of *An. koreicus*, all species were collected from these habitats. Only six species (*An. belenrae, An. kleini, An. lesteri, An. pullus, An. sinensis, An. sineroides*) were collected from irrigation ditches, ponds, ground pools, and uncultivated fields. Among the eight species, only *An. sinensis *was collected from all 10 habitat groups, while *An. kleini, An. pullus *and *An. sineroides *were sampled from all habitats, except artificial containers. The highest number of *Anopheles *larvae was found in the rice paddies (34.8%), followed by irrigation ditches (23.4%), ponds (17.0%), and stream margins, inlets and pools (12.0%) (Figure 4).

### Relationship among habitats and locations based on the species composition

**Figure 5:**
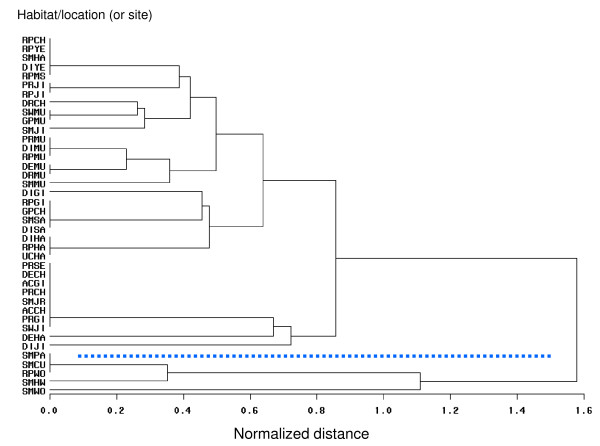
**Relationships, resulting from cluster analysis among the 40 *Anopheles*-positive habitat-locations (or sites) based on species composition**. Please see materials and methods section for details and abbreviations. Dashed line separates the upper cluster, which include all *Anopheles *sites, except 5 sites, from the lower cluster. Lower cluster includes stream margins, inlets and pools in Chungju nearby areas, Highway 517, Palgongsan Park and Woraksan Park, and rice paddies in Woraksan Park where *Anopheles *larvae were collected.

Cluster analysis for the 40 positive habitat sites (combined habitat and location variables), based solely on the species composition was conducted (Figure 5). The analysis resulted in two basal clusters, which reflect the dichotomy between the *An. lindesayi *habitats, the small lower cluster, and the habitats of the other seven *An*. species, the large upper cluster (Figure 5). The upper cluster includes the surface water habitats such as DE, ID, DR, GP, PR, RP, SM, SW, and UC, and AC. Only *An. sinensis *larvae were collected from the artificial containers, which were expected, considering that most *Anopheles *species in Hyrcanus, Barbirostris and Lindesayi Groups rarely breed in both natural and artificial containers [3]. The lower cluster includes only surface water habitats such as SM and RP. In this cluster, larvae of *An. lindesayi *were collected from two habitats (SM, RP) in three locations. In addition, *An. koreicus *and *An. sinensis *were found from SM habitats.

Like *An. sinensis*, the two major malaria vectors in the ROK, *An. kleini *and *An. pullus*, were commonly collected from rice paddies, irrigation ditches and ponds. Non-Hyrcanus Group species, such as *An. koreicus *and *An. lindesayi*, were usually collected from swamps, and stream margins (including stream inlets and pools), respectively. A previous study [6] indicated that overwintering larvae of *An. lindesayi *were found along the stream margins and stream pools of moderate to fast flowing streams, while first and second instars were collected in abundance in the late fall among shaded mountain stream eddies and margins. Some larvae (n = 66; 17% of total collected from all locations sampled) of *An. lindesayi *were also collected from rice paddies. However, those rice paddies were apparently not the primary habitats of this species. Sames *et al *[7] suggested that during heavy rains, stream pools may flood and some *An. lindesayi *larvae maybe washed down to rice-growing areas.

### Seasonal occurrence of *Anopheles *species from three locations

**Figure 6:**
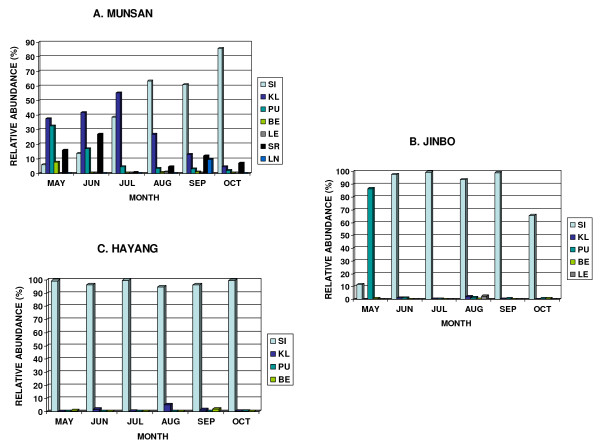
**Relative abundance of the *Anopheles (Anopheles) *larvae from various habitats in Munsan (A), Jinbo (B) and Hayang (C), Republic of Korea (May-October 2007)**. *An. belenrae*, BE; *An. kleini*, KL; *An. lesteri*, LE; *An. lindesayi*, LN; *An. pullus*, PU; *An. sinensis*, SI; *An. sineroides*, SR.

The results of the regular monthly surveys of mosquito larvae from three locations are shown in Figure 6(A-C). In Munsan areas (Figure 6A), a total of 4,615 *Anopheles *larvae belonging to seven species (*An. belenrae*, *An. kleini*, *An. lesteri*, *An. lindesayi*, *An. pullus*, *An. sinensis*, and *An. sineroides*) were collected from nine larval habitats (DE, DI, DR, GP, PR, RP, SM, SW, UC). *Anopheles kleini *was most abundant in early summer (May to July), whereas *An. sinensis *was the most frequently collected species in August, September and October. *Anopheles sinensis *and *An. lesteri *populations peaked in August, while *Anopheles pullus *peaked in May, and then decreased in June through October (Figure 6A).

In Jinbo areas (Figure 6B), a total of 1,897 *Anopheles *larvae from five species (*An. belenrae*, *An. kleini*, *An. lesteri*, *An. pullus*, and *An. sinensis*) were collected from five larval habitats (DI, PR, RP, SM, and SW). Overall, *An. pullus *was the most frequently collected species, followed by *An. sinensis, An. kleini, An*. *belenrae*, and *An. lesteri*. As in Munsan, *An. pullus *populations were highest in May, but in Jinbo the population peak of *An. kleini *was much later, occurring instead in August. *Anopheles sinensis *populations were high from June to October, with peak populations during August. *Anopheles kleini *populations peaked in October (Figure 6B).

In the Hayang area (Figure 6C), a total of 1,860 *Anopheles *larvae from four species (*An. belenrae*, *An. kleini*, *An. pullus*, and *An. sinensis*) were collected monthly from five larval habitats (DE, DI, RP, SW, and UC). *Anopheles sinensis *was the dominant species from May to October, followed by *An. kleini, An. belenrae *and *An. pullus*. Peak populations of *An. kleini, An. sinensis *and *An. belenrae *were in August, September and October, respectively, while *An. pullus *was collected only in October (Figure 6C).

**Figure 7:**
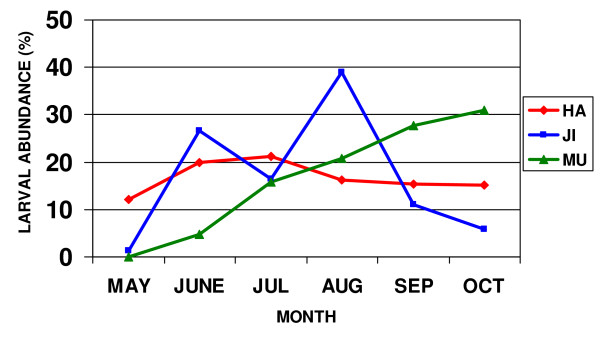
**Relative monthly abundance of *Anopheles sinensis *larvae from rice paddies and irrigation ditches in Munsan (MU), Jinbo (JI) and Hayang (HA), Republic of Korea (May-October 2007)**.

The eight *Anopheles (Anopheles) *species exhibited monthly fluctuations of larval density, depending on habitat and location. Abundance peaks varied between the three major locations sampled (Hayang, Jinbo and Munsan areas). For example, in rice paddies and irrigation ditches,*An. sinensis *was the most commonly collected species, with peak populations during July, August and October from Hayang, Jinbo, and Munsan, respectively (Figure 7). Sithiprasasna *et al *[22] also noted that *An. sinensis *(possibly *sensu lato*) was the most frequently collected mosquito, representing 97% of the specimens from various habitats sampled during August and September from two locations in the Gyeonggi Province, ROK.

### Relationship between normalized difference vegetation index (NDVI) data and mosquito larval population densities from various habitats in three locations

**Figure 8:**
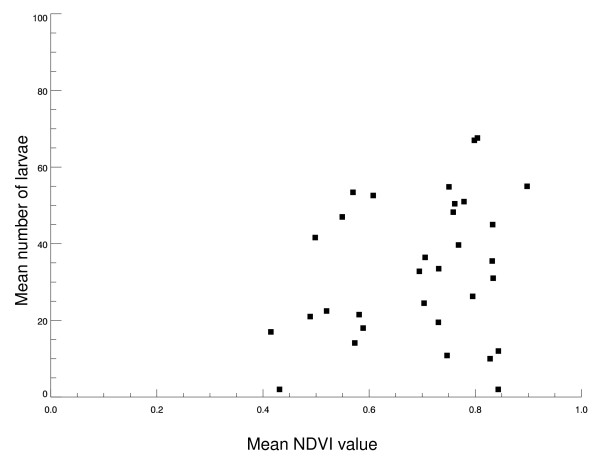
**Summary of the relationship between mean NDVI values and mean number of *Anopheles *larvae collected from various locations in the Republic of Korea**. Each point (solid square, n = 33 points) represents the average NDVI value for all collection locations within those sites for a single month (x-axis). This is plotted against the mean number of larvae for all collection locations within the site for that same month (y-axis).

A summary of the relationships between the NDVI values and *Anopheles *larval population densities shows that larval population densities increase as NDVI increases over the season (Figure 8). Furthermore, mosquito populations are likely to occur in large numbers when the ecological conditions are more homogenous over the landscape, e.g. in the middle of the summer when vegetation growth is maximal.

**Figure 9:**
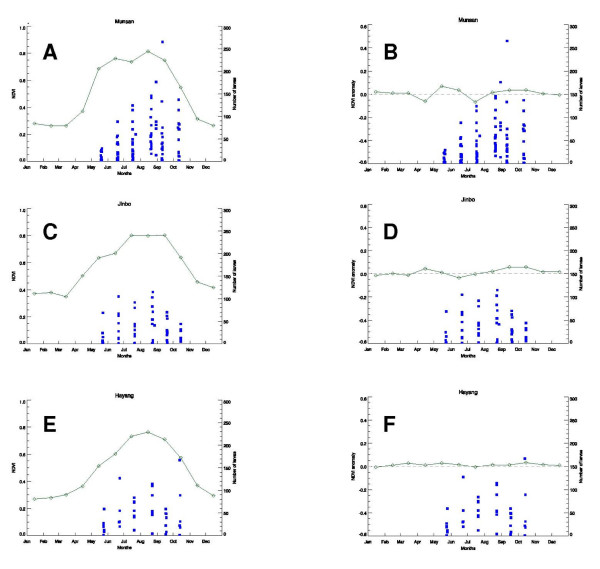
**Relationships between NDVI or NDVI anomalies and mean number of *Anopheles *larvae (solid squares) from Munsan (A, B), Jinbo (C, D), and Hayang (E, F)**. In A, C, E, NDVI values are represented by solid lines. In B, D, F, NDVI anomalies are represented by solid lines; mean NDVI values are shown in dashed lines.

In Munsan (Figure 9A), Jinbo (Figure 9C) and Hayang (Figure 9E), the NDVI began to increase in March and April with the start of the spring season, peaked in August, and then declined through the fall and into winter. Occurrence and abundance of mosquito larvae (solid squares, Figures 9A, C, E) followed the same pattern as the NDVI (solid line, Figures 9A, C, E), but with a lag of one or two months, increasing from May through August before declining. When the NDVI value for a given month is greater than the average, the NDVI anomaly (solid line) rises above the long-term average NDVI (dashed line) for Munsan (Figure 9B), Jinbo (Figure 9D), and Hayang (Figure 9F), respectively. The opposite happens when the NDVI for a given month is lower than the average. As shown in Figures 9B, D, E, the vegetative activity in the areas surrounding the collection sites for the months of May - October 2007 was neither abnormally dry nor abnormally wet, which indicated that 2007 was on average a normal year in the ROK. This can be observed by how closely the solid lines (NDVI anomaly values) follow the dashed lines (long-term average NDVI values). This suggests that the *Anopheles *larval population densities during the study period (May through October) in those three locations were not the result of abnormally dry or abnormally wet conditions.

Remote sensing data are commonly used to identify ecological conditions associated with vector-borne diseases especially mosquito vectors [8,23]. Most of these data were derived from measurements made by the Advanced High-Resolution Radiometer instrument aboard the National Oceanographic and Atmospheric Administration series of polar orbiting satellites. Measurements in the visible red and near infrared bands on this instrument are of specific relevance to ecology. The spectral signature of plant canopies is characterized by a strong chlorophyll absorption in the red portion of the spectrum and a very high reflectance in the near infrared portion. This unique spectral response of vegetation makes it possible to differentiate vegetation from other surface materials remotely. Derived NDVI values range between -1 to +1, with values below zero indicating absence of vegetation and those above zero showing increasing amounts of green vegetation. Precipitation and green vegetation dynamics are a major determinant of the life cycles of insects in a wide range of environments [9,24].

Remote sensing data are useful to identify conditions favorable for larval mosquito development, due to their preference for vegetated and humid areas. The distribution of mosquitoes is partly related to land use factors such as the presence or absence of wetlands, the type of surrounding vegetation, elevation and agricultural land use [8]. Many of these environmental factors can be mapped using remotely sensed data, and the normalized difference vegetation index (NDVI) can be used to explore or explain the relationship between mosquito population densities and vegetation/water seasonal patterns. Little is known about the use of remotely sensed data to estimate mosquito distributions in the ROK, except the initial work of Sithiprasasna *et al *[22]. Data on NDVI values and NDVI anomalies may be useful to predict the potential geographical distribution of *Anopheles *vectors and related species in the ROK. They may also be considered in developing ecological niche models for mosquito distributions [25,26], or to improve other existing statistical and related models.

In summary, the *Anopheles *larval populations fluctuated with the seasonal dynamics of vegetation for 2007. The peak in the mosquito populations coincided with the peak vegetative season in July - August (Figures 9A, C, E). During that period the landscape was more homogenous, creating more widespread conditions related to increased populations of mosquitoes over a much larger area than in the spring or the fall. However, in order to be able to accurately predict mosquito populations, the 2007 data are insufficient as they only reflect ordinary or average conditions. It is, therefore, necessary to have a sample of at least three to four years of mosquito population density data in order to compare the seasonal conditions and *Anopheles *populations over several years, and also to discern any differences that would provide predictive capability.

Finally, any geographical approach to sampling the natural habitats that includes all possible vector habitats, would lead to significant improvements of disease prevention and control programs [27]. Knowledge of *Anopheles *species composition from various breeding habitats, and the seasonal fluctuations of larval and adult populations from specific habitats and locations in the ROK, in addition to remote sensing data, will help in developing effective malaria and mosquito control strategies.

## Conclusions

The species composition of *Anopheles *larvae varied in different habitats at various geographical locations in the ROK. However, the higher numbers of species (seven out of eight) were collected from the rice paddies and streams (particularly margins, inlets and pool). Only *An. sinensis *larvae were collected from all ten habitat groups surveyed. Cluster analysis for the positive habitat sites (combined habitats and locations), based on species composition, showed two distinct basal clusters, with one cluster composed of *An. lindesayi *habitats, and the other cluster, the habitats of the other seven *An*. species. *Anopheles *larval populations fluctuated or increased with the seasonal dynamics of vegetation for 2007, as observed in Munsan, Jinbo and Hayang. Multi-year data of mosquito collections are required to provide a better characterization of the abundance of these insects from year to year which can potentially provide predictive capability of their population density based on remotely sensed ecological measurements.

## Competing interests

The authors declare that they have no competing interests.

## Authors' contributions

LMR, DHF, RCW and TAK designed the study. TLB was responsible for the implementation of the study in the laboratory, including PCR and sequencing of samples, and assisted by DHF and RCW. TAK, HCK and STC were responsible for the implementation of field collections, mosquito specimen sorting, processing, curating, and related activities. AA, MS and EPP were responsible for analysing data, including NDVI and NDVI anomalies. LMR analysed the data and drafted the manuscript, and also the principal investigator of the Global Emerging Infections Surveillance and Response Systems (GEIS) Research Project that funded this study. All authors read and approved the final manuscript.
